# Status Epilepticus among Older Adults in the United States

**DOI:** 10.3390/geriatrics4030045

**Published:** 2019-07-23

**Authors:** Priya Mendiratta, Neeraj Dayama, Jeanne Y Wei, Pallavi Prodhan, Parthak Prodhan

**Affiliations:** 1 Departments of Geriatrics, College of Medicine-University of Arkansas Medical Sciences, Little Rock, AR 72205, USA; 2 Department of Health Policy and Management, College of Public Health, University of Arkansas Medical Sciences, Little Rock, AR 72205, USA; 3 Pediatric Critical Care Medicine, College of Medicine-University of Arkansas Medical Sciences, Little Rock, AR 72205, USA

**Keywords:** older adults, status epilepticus, outcomes

## Abstract

Objective: This study aimed to identify temporal time trends and risk factors associated with mortality for hospitalized older adults with status epilepticus (SE). Design: A retrospective study was performed. Setting: Hospitalized patients were identified utilizing an administrative database—The Nationwide Inpatient Sample database from 1998 through September 2015. Patients: Patients were older adults 65 years and older with SE. Interventions: No interventions were undertaken. Measurements and Main Results: Demographic, temporal trends, clinical characteristics, and outcome data were abstracted. The results indicated that hospitalized elderly Americans with SE increased over the 11-year study period. Univariate and multivariate analyses were performed to evaluate risk factors associated with mortality in the study cohort. From the weighted sample, 130,109 subjects were included. Overall mortality was 19%. For age subgroups, the mortality was highest for the >85 years age group (24.1%) compared to the 65–75 years (19%) and 75–85 years (23%) age groups. Among investigated etiologies, the three most common causes of SE were acute ischemic stroke (11.2% of total) followed by non-traumatic brain hemorrhage (5.4%) and malignant brain lesions (4.9%). The highest mortality by etiology was noted for acute traumatic brain injury (TBI) (31.5%), non-traumatic brain hemorrhage (31%), and acute ischemic stroke (AIS) (30.1%). Multivariate analysis indicated that non-survivors when compared to survivors were more like to have the following characteristics: older age group, acute TBI, brain neoplasms, non-traumatic brain hemorrhage, AIS and central nervous system (CNS) infections, and utilization of mechanical ventilation. Associated conditions significantly increasing risk of mortality were sodium imbalance, cardiac arrest, anoxic brain injury, pneumonia, and sepsis. Comorbidities associated with increased risk of mortality included valvular heart disease, renal failure, liver disease, and neoplasms. Conclusions: The number of hospitalized elderly Americans with SE increased over the 11-year study period. Overall mortality was 19%, with even higher mortality among various patient subsets. Several demographic and co-morbid factors are associated with increased mortality in this age group.

## 1. Introduction

Status epilepticus (SE) is a life-threatening medical emergency characterized by prolonged seizure or multiple seizure with incomplete return to baseline. It is associated with a high morbidity and mortality of over 20% [1]. Careful history taking, neurological examination, and basic laboratory tests might identify the most common etiologies of SE. These include etiologies such as non-adherence or changes in anti-seizure therapies, drug-induced seizures, central nervous system infections, structural brain injury (acute or remote), acute metabolic imbalances, and alcohol withdrawal. In up to 20% of cases, this initial work-up may not reveal an obvious etiology [2].

Epidemiological studies indicate that older adults have an increased propensity for SE and associated higher mortality [3,4]. However, hospital-based studies in older adults are limited by their small sample size from single institutional studies [5,6,7,8,9,10,11]. Others included an all adult population [12] or were focused on specific subgroup etiologies [13]. Given the higher risk of a worse outcome in older adults, the risk factors for mortality in older adults and other clinical characteristics which are not well understood require further delineation. 

Therefore, the aim of this study was to investigate the clinical features, etiology, and outcomes of SE among older adults by using a large United States nationwide cohort of hospitalized patients.

## 2. Methods

### 2.1. Data and Sample

Retrospective data were pooled from the January 1998 to September 2015 Nationwide Inpatient Sample (NIS) databases, sponsored by the Agency for Healthcare Research and Quality (AHRQ) as part of the Healthcare Cost and Utilization Project (HCUP). NIS is the largest publicly available all-payer inpatient care database in the United States, representing a 20% stratified sample of all US community hospitals [14]. The NIS contains discharge level information from approximately eight million hospital stays from about 1000 non-federal hospitals and represents an approximately 20% stratified sample of all hospitals in the United States. It contains discharge level information for each patient including admission day, admission source, patient and hospital characteristics, discharge destination, and healthcare cost, and up to 15 diagnoses and procedures. A weighting variable provided by HCUP for trends and discharges was used to generate a national estimate [15]. Weighting the data allows nationally representative estimates to be produced. Additional details on the creation and use of weights can be found at https://www.hcup-us.ahrq.gov/databases.jsp.

### 2.2. Patient Population

All patients aged 65 years or older who had an *International Classification of Disease, 9th Revision, Clinical Modification* diagnosis code indicating status epilepticus (ICD-9-CM: 345.3) from 1998 through September 2015 were identified. Patients transferred to another short-term hospital were excluded in order to avoid double counting. 

### 2.3. Independent Variables and Outcomes

Variables studied included patient demographics (age, gender, race), hospital characteristics (rurality, region), insurance type, comorbidities, etiology, associated conditions, procedures, and outcomes. Study definitions and ICD-9-CM codes used to identify relevant conditions and procedures are provided in Supplemental Table S1. The study’s primary outcome was in-hospital mortality. 

### 2.4. Data Analyses

Patient and hospital characteristics are presented as relative frequencies and percentages for each independent variable. In order to analyze the overall trend of each independent variable during the study period, *t*-statistics were calculated. Trend *p* values were obtained. Univariate comparison between survivors and non-survivors was performed using *t*-test and chi-square test. Multivariate logistic regression was conducted for in-hospital mortality. All statistical analyses were performed using SAS 9.4^®^ (SAS Institute, Inc., Cary, NC, USA, 2013). All tests of significance were two-sided; significant results were defined at *p* < 0.05. Approval to conduct the study as exempt from human subjects review was received from the Institutional Review Board at the University of Arkansas for Medical Sciences. 

## 3. Results

### 3.1. Patient Characteristics

**Figure 1:**
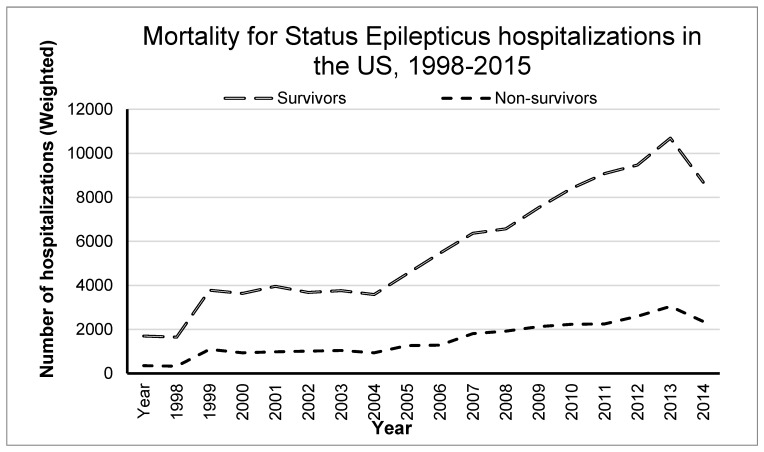
Temporal trends in status epilepticus hospitalization in the United States, 1998–September 2015.

From the weighted sample, 130,109 subjects with age 65 years and older were identified with a diagnosis of SE. Overall mortality was 19%. Figure 1 shows the increasing temporal trends for the SE cases and associated mortality over the study period.

Table 1 shows the clinical characteristics of the study population and compares survivors and non-survivors at hospital discharge. For age subgroups, the mortality was highest for the >85 years age group (24.1%) compared to the 65–75 years (19%) and 75–85 years (23%) age groups. Among investigated etiologies, the three most common causes of SE were acute ischemic stroke (11.2% of total) followed by non-traumatic brain hemorrhage (5.4%) and malignant brain lesions (4.9%). The highest mortality by etiology was noted for acute traumatic brain injury (TBI) (31.5%), non-traumatic brain hemorrhage (31%), and acute ischemic stroke (AIS) (30.1%). Among the associated conditions, the highest mortality was noted for cardiac arrest (74.6%), anoxic brain injury (68.2%), and sepsis (36.7%). Mortality for investigated co-morbid conditions was in the range of 24.1% to 31.6%. Continuous mechanical ventilation was noted in 46.9% of the cases, with a concomitant overall mortality of 33.1%.

Mortality for those mechanically ventilated for <96 and ≥96 consecutive hours was 26.2% and 41.2%, respectively. Given the increased mortality associated with a need for mechanical ventilation, subjects requiring continuous mechanical ventilation were compared to those not requiring continuous mechanical ventilation (Supplementary Table S2). 

### 3.2. Risk Factor Analysis for Mortality

Table 2 shows the univariate and multivariate analysis for evaluating risk factors associated with in-hospital mortality. Multivariate analysis indicates that non-survivors when compared to survivors were more like to have the following characteristics: older age group, acute TBI, brain neoplasms, non-traumatic brain hemorrhage, AIS, central nervous system (CNS) infections, and utilization of mechanical ventilation. Associated conditions significantly increasing risk of mortality were sodium imbalance, cardiac arrest, anoxic brain injury, pneumonia, and sepsis. Comorbidities associated with increased risk of mortality included valvular heart disease, renal failure, liver disease, and neoplasms of any location (see Table 2 for odds ratio and 95% confidence intervals).

## 4. Discussion

To our knowledge, this is the largest nationwide cohort study of hospitalized SE in older adults. The strengths of this study lie primarily in its size and generalizability. In contrast to previous single-center studies, analysis of patients from a nationwide cohort allows us to investigate outcomes for a much more diverse and representative sample. 

Our study confirms results from prior epidemiological studies on SE that indicated higher mortality with increasing age. Our study indicates a mortality of 24.1% for the ≥85 years age group compared to 19% in the 65–75 years age group. Higher mortality in older adults also contrasts to the less than 5% mortality among younger adults well into the fourth decade of life [11,16]. Previously, among older adults, the overall mortality rates with SE in hospital-based studies have ranged from 6.5% to 31% [5,6,7,8,9,10,11]. However, in contrast to our study in older adults, these prior investigations among older adults were limited by being single center studies, primarily done outside the United States, and with very small sample sizes (range of 33 to 140 subjects) [5,6,7,8,9,10,11]. 

However, the overall mortality rate of 19% in older adults with SE in our study masks the higher mortality rates in subgroup populations such as those with anoxic brain injury (68%), cardiac arrest (75%), and other groups with >30% mortality (TBI, non-traumatic hemorrhage, those with pneumonia, sepsis, coagulopathy, renal failure, pulmonary circulation disease, AIS, and those requiring continuous mechanical ventilation for >96 h). Many of these etiologies, comorbid and associated conditions are also independently associated with increased mortality. Similar data specific to older adults is currently limited. Villella et al. [9], in their single-center study on 90 subjects over 70 years of age, reported a 31.1% mortality and identified SE duration of >12 h, modified Status Epilepticus Severity Score (mSTESS) predictive score, and development of complications as risk factor associated with mortality. Other centers with reported lower mortality in their single center studies have identified acute presentation [5] refractory SE [8], and abnormal hyperintensities in diffusion weighted images [8]. Our database-based study is limited in its inability to calculate mSTESS scores and in identifying specific imaging findings. Given the differences in outcomes based on underlying diagnosis, our results may help clinical practitioners with better prognosticating outcomes in older adults.

The duration of mechanical ventilation has been previously used as a surrogate for severity of SE [17,18] to identify sicker sub-groups among patients with SE [12]. Those receiving mechanical ventilation for longer durations are considered to have refractory and super-refractory SE [17,18]. In our study, mortality was 41.3% among those with continuous mechanical ventilation for greater than 96 h compared to 26.5% in those with MV less for than 96 h and 10.8% in those not mechanically ventilated. Being mechanically ventilated significantly increased the odds of mortality in older adults. Similar to our results, Sánchez Fernandez et al. [17] analyzed the NIS database from year 2007 to 2012 and observed higher costs and mortality rates of 8.7%, 15.5%, and 30.2% for those receiving no MV, MV for <96 h, and MV for >96 h, respectively, in the older adult subgroup. However, Sánchez Fernandez et al. [17] were primarily looking at costs and did not provide a detailed analysis of risk factors specific to older adults with SE. Similarly, Strzelczyk et al. [18] reported mortality rates of 9.6%, 15%, and 39.9% for those receiving no MV, MV for <48 h, and MV for >48 h, respectively, in an all-adults German database study.

The strength of this study lies primarily in its size and generalizability. In contrast to previous single-center studies. The ability to control for the severity of subarachnoid hemorrhage using the Nationwide Inpatient Sample Subarachnoid Severity Scale also allowed for more robust modeling. By selecting only patients who received definitive therapy for their aneurysm, we attempted to minimize the inclusion of patients with alternate etiologies of subarachnoid hemorrhage (for example, traumatic), as well as the confounding effect of re-bleeding in untreated patients or patients who were transitioned to comfort care before aneurysmal control. 

There are several limitations in the current study. First, the NIS database is an administrative database and lacks detailed information on clinical examination, medications use, laboratory, imaging results, and any after-hospital discharge data. Additionally, such large, database-driven observational studies are inherently susceptible to coding errors and selection bias. The accuracy of ICD-9-CM coding is an inherent limitation to this type of study. However, the Healthcare Cost and Utilization Project quality control measures should minimize these possibilities. The NIS contains discharge-level and not patient-level records. Consequently, individuals who are hospitalized for SE multiple times in a year may be represented multiple times in NIS. Exclusion of patients transferred to another short-term hospital in order to avoid double counting could result in missing cases. However, the large sample size of the weighted sample should minimize the impact of missed cases on study outcomes. Known clinical predictors of seizures, such as the presence and duration of loss of consciousness, or severity and extent of injury, could not be assessed in this study. Non-convulsive SE, a condition associated with high mortality rates, was excluded due to lack of proper ICD-9 diagnosis codes and the clinical implications of this entity remain poorly defined. The results of this study must be interpreted in the context of the study design. The ICD-9 codes for mechanical ventilation only allow for classification based on the 96-hour cutoff and thus are limited to provide more granular data to evaluate mechanical ventilation. Given the lack of specific ICD-9 codes, it is not possible to assess whether clinical decision on limiting escalation of care and comfort care accounted for higher mortality in older age groups. The journal constraints have precluded us from elaborating in the discussion on various subsets of patients in more detail. We plan on elaborating these subsets in future publications.

The NIS is an administrative database that aims to gather data for billing purposes and can be limited by erroneous coding. However, the Healthcare Cost and Utilization Project quality control measures should minimize these possibilities. Furthermore, the hard clinical end-points used in our analysis are more difficult to miscode. Nevertheless, the potential exists for unmeasured confounders that may bias the outcomes. 

## 5. Conclusions

We identified numerous risk factors for in-hospital mortality, which include underlying comorbid factors related to multiple organ systems and acute presentations like cardiac arrest anoxic brain injury, and TBI, with a higher risk attributed to heart failure, renal failure, and liver dysfunction. Our study indicated risk factors for death amongst a host of additional comorbid conditions and complications, such as cardiac arrest and anoxic brain injury.

## Figures and Tables

**Table 1:** Comparison of survivors and non-survivors at hospital discharge for older adults with status epilepticus.

Variables	Total	Survivors	Non-Survivors (*n*, %)	*p*-Value
		(*n*, %)		
	***N* = 130,109**	***N* = 102,540**	***N* = 27,569**	
**Age, years**				
*65 to 74*	63,227	51,206 (80.91)	12,021 (19.09)	<0.001
*75 to 84*	47,105	36,289 (77.04)	10,815 (22.96)	<0.001
*85 and above*	19,778	15,045 (76.07)	4734 (23.93)	<0.001
*Female gender*	74,627	59,469 (79.69)	15,158 (20.31)	<0.001
**Race**				
*White*	70,383	54,805 (77.87)	15,578 (22.13)	0.001
*Non-white*	59,727	47,735 (79.92)	11,991 (20.08)	0.001
*Black*	27,596	22,708 (82.29)	4887 (17.71)	<0.001
Hispanic	8073	6331 (78.42)	1743 (21.58)	0.699
*Other race*	3135	2322 (71.07)	813 (28.93)	0.002
Missing race	17,934	14,173 (79.03)	3762 (20.97)	0.764
**Hospital type**				
*Rural*	12,328	10,348 (83.94)	1980 (16.06)	<0.001
*Urban non-teaching*	48,679	39,130 (80.38)	9548 (19.62)	<0.001
*Urban teaching*	68,606	52,701 (76.82)	15,904 (23.18)	<0.001
**Hospital location**				
*Northeast*	26,981	20,865 (77.33)	6115 (22.67)	0.01
*Midwest*	27,014	21,760 (80.55)	5254 (19.45)	0.002
*South*	51,788	41,421 (79.98)	10,366 (20.02)	0.001
*West*	24,327	18,493 (76.02)	5834 (23.98)	<0.001
**Insurance type**				
*Medicare*	114,256	90,670 (79.36)	23,586 (20.64)	<0.001
Medicaid	3453	2802 (81.15)	651 (18.85)	0.129
*Private*	9666	7141 (73.88)	2525 (26.12)	<0.001
Uninsured	787	585 (74.33)	202 (25.67)	0.188
**Etiology**				
*Acute traumatic brain injury*	4777	3268 (68.41)	1508 (31.59)	<0.001
Malignant brain neoplasm	6316	5056 (80.05)	1260 (19.95)	0.275
*Benign brain neoplasm*	1915	1735 (90.60)	180 (9.40)	<0.001
Arterio-venous malformation	215	190 (88.37)	25 (11.62)	0.119
*Non-traumatic brain hemorrhage*	6988	4822 (69.00)	2166 (31.00)	<0.001
*Epilepsy*	4949	4398 (88.87)	551 (11.13)	<0.001
*Acute ischemic stroke*	14,556	10,174 (69.90)	4381 (30.10)	<0.001
*Central nervous system infections*	4605	3284 (71.31)	1321 (28.69)	<0.001
**Associated conditions**				
*Sodium imbalance*	22,507	16,839 (74.82)	5668 (25.18)	<0.001
*Anoxic brain injury*	14,365	4573 (31.83)	9792 (68.17)	<0.001
*Cardiac arrest*	9403	2384 (25.35)	7019 (74.65)	<0.001
*Pneumonia*	20,089	13,769 (68.54)	6320 (31.46)	<0.001
*Sepsis*	21,219	13,426 (63.27)	7793 (36.73)	<0.001
*Coagulopathy*	10,804	7377 (68.28)	3428 (31.72)	<0.001
**Comorbidity**				
*Elixhauser mortality score*	11.87 (23.42)	10.85 (23.01)	15.64 (23.40)	<0.001
*Congestive heart failure*	23,861	17,227 (72.20)	6634 (27.80)	<0.001
*Valvular disease*	7268	5343 (73.51)	1924 (26.49)	<0.001
*Pulmonary circulation disease*	3973	2718 (68.41)	1256 (31.59)	<0.001
*Chronic pulmonary disease*	27,515	20,965 (76.19)	6550 (23.81)	<0.001
*Diabetes w/ chronic complications*	6550	4797 (73.24)	1753 (26.76)	<0.001
*Renal failure*	20,546	14,429 (70.23)	6117 (29.77)	<0.001
*Liver disease*	2964	2165 (73.04)	799 (26.96)	0.001
*Neoplasms (all body locations)*	61,740	46,839 (75.86)	14,901 (24.14)	<0.001
**Procedures**				
Tracheostomy	6341	4980 (78.54)	1362 (21.46)	0.802
*Gastrostomy tube placement*	9773	8589 (87.88)	1184 (12.12)	<0.001
*Mechanical ventilation*	61,023	40,834 (66.92)	20,189 (33.08)	<0.001
Unspecified duration	138	105 (76.09)	33 (23.91)	0.728
*Less than 96 consecutive hours*	32,944	24,299 (73.76)	8644 (26.24)	<0.001
*96 consecutive hours or more*	27,942	16,430 (58.80)	11,512 (41.20)	<0.001
**Outcomes**				
*Hospital length of stay, days*	10.36 (27.12)	10.85 (23.01)	15.64 (23.40)	<0.001
*mean (std)*				

**Table 2:** Univariate and multivariate analysis for risk factors associated with in-hospital mortality for older adults with status epilepticus.

	Univariate				Multivariate			
Variables	Odds Ratio	95% Confidence Interval		*p*-Value	Odds Ratio	95% Confidence Interval		*p*-Value
**Age, years**								
65–74	0.78	0.73	0.82	0.000	Reference			
75–84	1.18	1.11	1.25	0.000	1.40	1.30	1.51	0.0000
≥85	1.21	1.11	1.30	0.000	1.87	1.68	2.07	0.0000
Female gender	0.88	0.83	0.94	0.000	1.05	0.97	1.12	0.218
**Etiology**								
Acute traumatic brain injury	1.76	1.53	2.02	0.000	2.67	2.27	3.13	0.0000
Malignant neoplasm brain	0.92	0.80	1.07	0.276	1.56	1.31	1.86	0.0000
Benign neoplasm brain	0.38	0.27	0.54	0.000	0.61	0.41	0.91	0.015
Arterio-venous malformation	0.48	0.19	1.23	0.127	0.96	0.32	2.81	0.933
Non-traumatic brain hemorrhage	1.73	1.53	1.95	0.000	2.33	2.01	2.69	0.0000
Epilepsy	0.46	0.37	0.56	0.000	0.53	0.43	0.66	0.0000
Acute ischemic stroke	1.72	1.57	1.87	0.000	2.07	1.86	2.29	0.0000
Central nervous system infection	1.52	1.31	1.77	0.000	1.76	1.48	2.10	0.0000
**Associated conditions**								
Sodium imbalance	1.32	1.22	1.42	0.000	1.11	1.00	1.23	0.045
Anoxic brain injury	11.80	10.82	12.87	0.000	5.83	5.18	6.56	0.0000
Cardiac arrest	14.35	12.84	16.02	0.000	4.71	4.05	5.49	0.0000
Pneumonia	1.92	1.78	2.07	0.000	1.29	1.16	1.42	0.0000
Sepsis	2.62	2.43	2.82	0.000	2.04	1.85	2.25	0.0000
Coagulopathy	1.83	1.66	2.02	0.000	1.02	0.89	1.17	0.769
**Co-morbidities**								
Elixhauser mortality score	1.04	1.04	1.04	0.000	1.03	1.02	1.04	0.0000
Congestive heart failure	1.57	1.46	1.69	0.000	0.93	0.84	1.04	0.19
Valvular heart disease	1.36	1.21	1.54	0.000	1.24	1.07	1.43	0.005
Pulmonary circulation disease	1.75	1.51	2.04	0.000	1.02	0.83	1.24	0.872
Chronic lung disease	1.21	1.13	1.30	0.000	1.00	0.92	1.09	0.946
Diabetes mellitus with complications	1.38	1.22	1.57	0.000	1.27	1.09	1.49	0.003
Renal failure	1.74	1.62	1.88	0.000	1.12	1.02	1.24	0.023
Liver failure	1.38	1.15	1.66	0.000	1.35	1.09	1.69	0.007
Neoplasms (all locations)	1.40	1.32	1.48	0.000	0.71	0.64	0.78	0.0000
**Procedures**								
Tracheostomy	1.02	0.89	1.17	0.802	0.40	0.33	0.50	0.0000
Gastrostomy tube placement	0.49	0.43	0.57	0.000	0.26	0.22	0.32	0.0000
No mechanical ventilation	4.13	3.86	4.42	0.000	Reference			
Consecutive mechanical ventilation, unspecified	1.34	0.59	3.06	0.482	1.33	0.45	3.92	0.608
Consecutive mechanical ventilation, <96 h	1.50	1.41	1.60	0.000	1.72	1.58	1.88	0.0000
Consecutive mechanical ventilation, >96 h	3.76	3.52	4.01	0.000	3.02	2.73	3.35	0.0000

## Supplementary Materials

The following are available online at https://www.mdpi.com/2308-3417/4/3/45/s1, Table S1: Codes and classification for variables included in the study, Table S2: Additional demographic and hospital characteristics not included in multivariate analysis.
