# Supplementary material for: Status Epilepticus among Older Adults in the United States
Source: Geriatrics (Basel). 2019 Jul 23;4(3):45. doi: 10.3390/geriatrics4030045 (PMC6787625; doi:10.3390/geriatrics4030045)
Supplement: Supplementary file 1 [file geriatrics-04-00045-s001.pdf]

Supplementary Materials

Status Epilepticus among Older Adults in the United States

Priya Mendiratta MD, MPH[1*], Neeraj Dayama, MD[2*], Jeanne Y Wei, MD, PhD[1], Pallavi Prodhan [1], and Parthak Prodhan MD[3]

Departments of Geriatrics[1], College of Medicine-University of Arkansas Medical Sciences, Little Rock, Arkansas; Department of Health Policy and Management[2] College of Public Health, University of Arkansas Medical Sciences, Little Rock, Arkansas, Pediatric Critical Care Medicine, College of Medicine-University of Arkansas Medical Sciences, Little Rock, Arkansas

* Contributed equally to the study

Correspondence: Priya Mendiratta, MD, MPH, Associate Professor in Geriatrics, Department of Geriatrics, Reynolds Institute on Aging, College of Medicine, University of Arkansas Medical Sciences, 4301 W. Markham Street, Little Rock, AR 72205; Tel: (501)-658-3320; Fax (501)-603-1646

Supplementary Table 1: Codes and classification for variables included in the study.

Variables	CCS Code	ICD-9 codes
**Status epilepticus**		345.3
**Brain Co-morbidities**		
Acute Traumatic brain injury	233	
Malignant neoplasm of the brain		191x, 192.1, 192.8, 198.3, 198.4, 200.5
Benign neoplasm of the brain		225.0, 225.1, 225.2
Arterio-venous malformation		747.81
Non traumatic brain hemorrhage		430, 431
Subdural-hematoma		432.1, 852.2, 852.3
Epilepsy		[illegible]5.0x, 345.1x, 345.4x, 345.5x, 345.8x, 345.9x
Acute ischemic stroke		[illegible]3.01, 433.11, 433.21, 433.31, 433.81, 433.91, 434.01, 434.11, 434.91, 436
Central nervous system infections	76,77,78	
Cardiac arrest	107	
Sodium imbalance		276.1, 276.0
Anoxic brain injury		348.1
Comorbid conditions		From Elixhauser co-morbidity index components https://hcup-us.ahrq.gov/toolssoftware/comorbidity/comorbidity.jsp
**Procedures**		
Intubation and mechanical ventilation		96.04, 96.70, 96.71, 96.72
Tracheostomy		31.1, 31.21, 31.29

Percutaneous endoscopic gastrostomy –tube placement		43.11

Classification of hospital: Hospitals were classified as per NIS guidelines

BEDSIZE CATEGORIES (Beginning in 1998)			
Location and Teaching Status	Hospital Bedsize		
	Small	Medium	Large
**NORTHEAST REGION**			
Rural	1-49	50-99	100+
Urban, nonteaching	1-124	125-199	200+
Urban, teaching	1-249	250-424	425+
**MIDWEST REGION**			
Rural	1-29	30-49	50+
Urban, nonteaching	1-74	75-174	175+
Urban, teaching	1-249	250-374	375+
**SOUTHERN REGION**			
Rural	1-39	40-74	75+
Urban, nonteaching	1-99	100-199	200+
Urban, teaching	1-249	250-449	450+
**WESTERN REGION**			
Rural	1-24	25-44	45+
Urban, nonteaching	1-99	100-174	175+
Urban, teaching	1-199	200-324	325+

Supplementary Table 2: Additional demographic and hospital characteristics not included in multivariate analysis

Variables	Odds Ratio	95% Conf. Interval		p- value
Race				
White	1.13	1.06	1.2	0
Non white	0.88	0.83	0.94	0
Black	0.76	0.7	0.82	0
Hispanic	1.03	0.9	1.17	0.7
Other race	1.31	1.1	1.56	0.002
Missing race	0.99	0.89	1.09	0.764
Hospital type				
Rural	0.69	0.61	0.78	0
Urban-Non teaching	0.86	0.8	0.92	0
Urban teaching	1.29	1.21	1.38	0
Hospital location				
Northeast	1.12	1.03	1.21	0.011
Midwest	0.87	0.8	0.95	0.002
South	0.89	0.83	0.95	0.001
West	1.22	1.13	1.32	0
Insurance type				
Medicare	0.78	0.71	0.85	0
Medicaid	0.86	0.71	1.04	0.13
Private	1.35	1.21	1.5	0
Uninsured	1.29	0.88	1.88	0.189

Supplementary Tables 3: Comparison of groups with and without mechanical ventilation (MV).

	**Total**	**No MV**	**Received MV**	**p-value**
	**N = 130,109**	**N = 69,086**	**N = 61,023**	
**Age, years**				
65-74	63,227	31,695 (45.88)	31,531 (51.67)	<0.000
75-84	47,105	24,791 (35.89)	22,313 (36.56)	0.253
>= 85	19,778	12,599 (18.24)	7,179 (11.76)	<0.000
Female gender	74,627	41,364 (59.87)	33,263 (54.51)	<0.000
Race				
White	70,383	37,904 (54.86)	32,479 (53.22)	0.014
Non white	59,727	31,182 (45.14)	28,545 (46.78)	0.014
Black	27,596	13,716 (19.85)	13,880 (22.74)	<0.000
Hispanic	8,073	3,888 (5.63)	4,186 (6.86)	<0.000
Other race	3,135	1,414 (2.05)	1,721 (2.82)	0.000
Missing race	17,934	10,700 (15.49)	7,234 (11.85)	<0.000
Hospital type				
Rural	12,328	9,259 (13.40)	3,070 (5.03)	<0.000
Urban-non teaching	48,679	27,298 (39.51)	21,381 (35.04)	<0.000
Urban teaching	68,606	32,312 (46.77)	36,294 (59.48)	<0.000
Hospital location				
Northeast	26,981	13,947 (20.19)	13,033 (21.36)	0.048
Midwest	27,014	14,679 (21.25)	12,334 (20.21)	0.080
South	51,788	27,776 (40.21)	24,012 (39.35)	0.246
West	24,327	12,683 (18.36)	11,644 (19.08)	0.209
Insurance type				
Medicare	114,256	60,994 (88.29)	53,262 (87.28)	0.016
Medicaid	3,453	1,605 (2.32)	1,848 (3.03)	0.001
Private	9,666	5,033 (7.28)	4,633 (7.59)	0.345
Uninsured	787	344 (0.50)	444 (0.73)	0.018
**Etiology**				
Acute traumatic brain injury	4,777	1,932 (2.80)	2,845 (4.66)	<0.000
Malignant neoplasm brain	6,316	3,871 (5.60)	2,445 (4.01)	<0.000
Benign neoplasm brain	1,915	1,033 (1.50)	882 (1.45)	0.744
Arterio-venous malformation	215	111 (0.16)	104 (0.17)	0.827
Non-traumatic brain hemorrhage	6,988	2,872 (4.16)	4,116 (6.74)	<0.000
Epilepsy	4,949	2,766 (4.00)	2,183 (3.58)	0.079
Acute ischemic stroke	14,556	6,590 (9.54)	7,966 (13.05)	<0.000
Central nervous system infection	4,605	1,785 (2.58)	2,820 (4.62)	<0.000
**Associated conditions**				
Sodium imbalance	22,507	9,784 (14.16)	12,723 (20.85)	<0.000
Anoxic brain injury	14,365	1,602 (2.32)	12,763 (20.92)	<0.000
Cardiac arrest	9,403	728 (1.05)	8,675 (14.22)	<0.000

Pneumonia	20,089	6,252 (9.05)	13,837 (22.68)	<0.000
Sepsis	21,219	6,786 (9.82)	14,433 (23.65)	<0.000
Coagulopathy	10,804	3,971 (5.75)	6,833 (11.20)	<0.000
**Co-morbidities**				
Elixhauser mortality score				
Congestive heart failure	23,861	10,565 (15.29)	13,297 (21.79)	<0.000
Valular heart disease	7,268	3,709 (5.37)	3,558 (5.83)	0.112
Pulmonary circulation disease	3,973	1,482 (2.15)	2,491 (4.08)	<0.000
Chronic lung disease	27,515	12,620 (18.27)	14,895 (24.41)	<0.000
Diabetes mellitus with complications	6,550	3,000 (4.34)	3,551 (5.82)	<0.000
Renal failure	20,546	8,947 (12.95)	11,599 (19.01)	<0.000
Liver failure	2,964	1,272 (1.84)	1,692 (2.77)	<0.000
Neoplasms (all body locations)	61,740	27,147 (39.30)	34,593 (56.69)	<0.000
**Procedures**				
Tracheostomy	6,341	364 (0.53)	5,977 (9.79)	<0.000
Gastrostomy tube	9,773	3,272 (4.74)	6,501 (10.65)	<0.000
**Mortality**	27,569	7,380 (10.68)	20,189 (33.08)	<0.000
**Discharge status**				
Home health care	14,246	9,274 (13.42)	4,972 (8.15)	<0.000
Routine	22,908	16,530 (23.93)	6,378 (10.45)	<0.000
Skilled nursing facility	58,574	32,526 (47.08)	26,047 (42.68)	<0.000
